# Polyamine-mediated crosstalk in plant–microbe systems: implications for stress tolerance and crop protection

**DOI:** 10.3389/fpls.2026.1913716

**Published:** 2026-09-14

**Authors:** Younes Rezaee Danesh, Emanuele La Bella, Claudio Cannata, Alessandro Vitale

**Affiliations:** 1 Department of Plant Protection, Faculty of Agriculture, Van Yuzuncu Yil University, Van-Türkiye, Türkiye; 2 Department of Agriculture, Food and Environment, University of Catania, Catania, Italy

**Keywords:** crop protection, defense mechanisms, plant-microbe interactions, polyamines, reactive oxygen species (ROS), stress tolerance

## Abstract

The interaction between plants and microbes is a complex network of signaling pathways that plays a pivotal role in plant health and resilience to environmental stressors. Recently, polyamines (PAs), including putrescine, spermidine, and spermine, have emerged as crucial mediators in the crosstalk between plants and microbes. These small molecules are not only involved in cellular growth and differentiation but also significantly influence the plant’s response to biotic and abiotic stresses. Research indicates that PAs can enhance the expression of defense-related genes, thereby increasing resistance to pathogens such as *Pseudomonas syringae* and *Hyaloperonospora arabidopsidis*. Furthermore, PAs interact with reactive oxygen species (ROS) to form positive feedback loops that amplify defensive responses during pathogen attacks. Additionally, the modulation of polyamine metabolism has been shown to improve crop resilience under stress conditions, suggesting their potential as biostimulants in agricultural practices. This review explores the multifaceted roles of PAs in plant-microbe interactions, highlighting their implications for stress tolerance and crop protection. Understanding the molecular mechanisms underpinning polyamine-mediated crosstalk could lead to the development of innovative strategies for enhancing crop productivity and sustainability in the face of increasing environmental challenges.

## 1 Introduction

The role of PAs in plant–microbial interactions has gained increasing recognition, emerging as an essential and immensely significant aspect that effectively guides the development of sustainable and environmentally friendly agricultural practices. Recent studies underscore the necessity for plants to maintain a strict polyamine homeostasis, which is physiologically and developmentally regulated, in order to co–optimize their growth, development, and resilience to numerous stress factors (Chen et al., 2019; Seifi and Shelp, 2019). All types of plant–microbial interactions—whether beneficial, pathogenic, or neutral—profoundly influence the extent and type of changes that are observed in the levels of PAs within plants (Valdés-Santiago and Ruiz-Herrera, 2014; Takahashi, 2020). Furthermore, the varying concentrations of PAs encountered under diverse plant conditions and during specific microbial interactions are pivotal in governing differential signaling responses and the selection of target plant genes in a highly partner-specific manner (Seifi and Shelp, 2019). Therefore, delving into the intricate complexities of plant–microbial interactions represents a notably promising and stimulating avenue that can lead to invaluable insights regarding the elaborate nature of polyamine functions within various plants. Compelling evidence gathered from ongoing research robustly suggests that PAs are integral to orchestrating a multitude of interactions that take place between plants and microorganisms (Chen et al., 2019; Takahashi, 2020). It is evident that these biological functions possess both fundamental biological importance and significant agronomic implications for the agricultural sector at large. Moreover, the active search for soluble microbial metabolites and microbial-derived physiological-compatible polyamine analogs was initially driven, from a chemical perspective, by the well-documented and established reciprocal interaction that exists within the realm of organic chemistry, particularly between classical PAs and various water-soluble nutrients (Valdés-Santiago and Ruiz-Herrera, 2014). This includes well-known vitamins such as vitamin D, vitamin B, and vitamin B2, all of which are essential to human health as well. The role of PAs extends significantly to enhancing the ability of certain plant species, especially those of considerable agricultural and agronomic importance, to absorb exogenous water-soluble saturated and unsaturated organic PAs through their leaf stomata and root tissues with remarkable efficiency (Pál et al., 2015; Chen et al., 2019). Some of these plant species are capable of effectively taking up vital light energy through their various bodily structures, exhibiting remarkable biotic symbiotic associations with both beneficial and pathogenic fungi, as well as bacteria (Seifi and Shelp, 2019). This unique ability enables them to favorably integrate and co-assimilate foreign biogenic nutrients derived from decomposed organic matter, thereby enriching their nutritional profiles significantly. This phenomenon represents a significant and fertile area for investigation into PAs, further illuminating the intricate and multifaceted complexities discovered within the highly interconnected web of plant–microorganism interactions. These interactions play essential roles in maintaining ecological balance, thereby demonstrating the necessity and utility of PAs in enhancing not only plant health and productivity but also the sustainability of agricultural practices (Liu et al., 2015; Pál et al., 2015; Takahashi, 2020).

## 2 Polyamines: biosynthesis, regulation, and functions in plants

The major PAs in plants include putrescine (Put), spermidine (Spd), and spermine (Spm), which are the most extensively studied because of their central roles in plant growth, development, and responses to biotic and abiotic stresses (Minocha et al., 2014; Chen et al., 2019). In addition, several other PAs, including thermospermine, cadaverine, norspermidine, and homospermidine, have been identified in particular plant species or taxonomic groups, although their physiological functions and distribution remain comparatively less understood (Takahashi and Kakehi, 2010; Kusano and Suzuki, 2015). Therefore, this review focuses primarily on putrescine, spermidine, and spermine because they represent the predominant PAs involved in plant–microbe interactions and stress responses. These compounds are intricately involved in a myriad of essential biological functions, such as cell division, the process of differentiation, flowering, the development of fruits, and the onset of senescence (Chen et al., 2019; Takahashi, 2020). Moreover, the importance of PAs in mediating responses to both abiotic and biotic stresses has been extensively documented and recognized within the scientific community (Liu et al., 2015; Seifi and Shelp, 2019). The biosynthesis, catabolism, and major physiological functions of PAs in plants are summarized in **Figure 1**. Polyamine metabolism involves tightly regulated biosynthetic and catabolic pathways that control the cellular levels of putrescine, spermidine, and spermine and generate important signaling metabolites including GABA (Sobieszczuk-Nowicka and Legocka, 2014; Chen et al., 2019). Putrescine is synthesized through two alternative pathways. In the ornithine pathway, ornithine is directly decarboxylated by ornithine decarboxylase (ODC) to produce putrescine. In contrast, the arginine pathway is a multistep process in which arginine is first converted to agmatine by arginine decarboxylase (ADC), followed by conversion to N-carbamoylputrescine via agmatine iminohydrolase (AIH), and finally to putrescine by N-carbamoylputrescine amidohydrolase (CPA). Unlike many bacteria, higher plants predominantly synthesize putrescine from arginine through the sequential activities of ADC, AIH, and CPA rather than via a direct agmatinase-mediated pathway (Kusano et al., 2008; Takahashi and Kakehi, 2010). Putrescine is subsequently converted into spermidine and spermine through S-adenosylmethionine decarboxylase (SAMDC)-dependent reactions catalyzed by spermidine synthase (SPDS) and spermine synthase (SPMS). Polyamine catabolism, mediated by polyamine oxidases (PAO), generates important signaling molecules, including hydrogen peroxide (H_2_O_2_) and γ-aminobutyric acid (GABA), which contribute to redox regulation and stress signaling. Beyond these metabolic pathways, **Figure 1** also summarizes the major physiological roles of PAs in plant growth and development, gene regulation, membrane stability, root architecture, and adaptation to abiotic and biotic stresses. This dynamic balance between biosynthesis and catabolism maintains cellular homeostasis and provides the metabolic foundation for the signaling functions of PAs discussed in the following sections.

**Figure 1:**
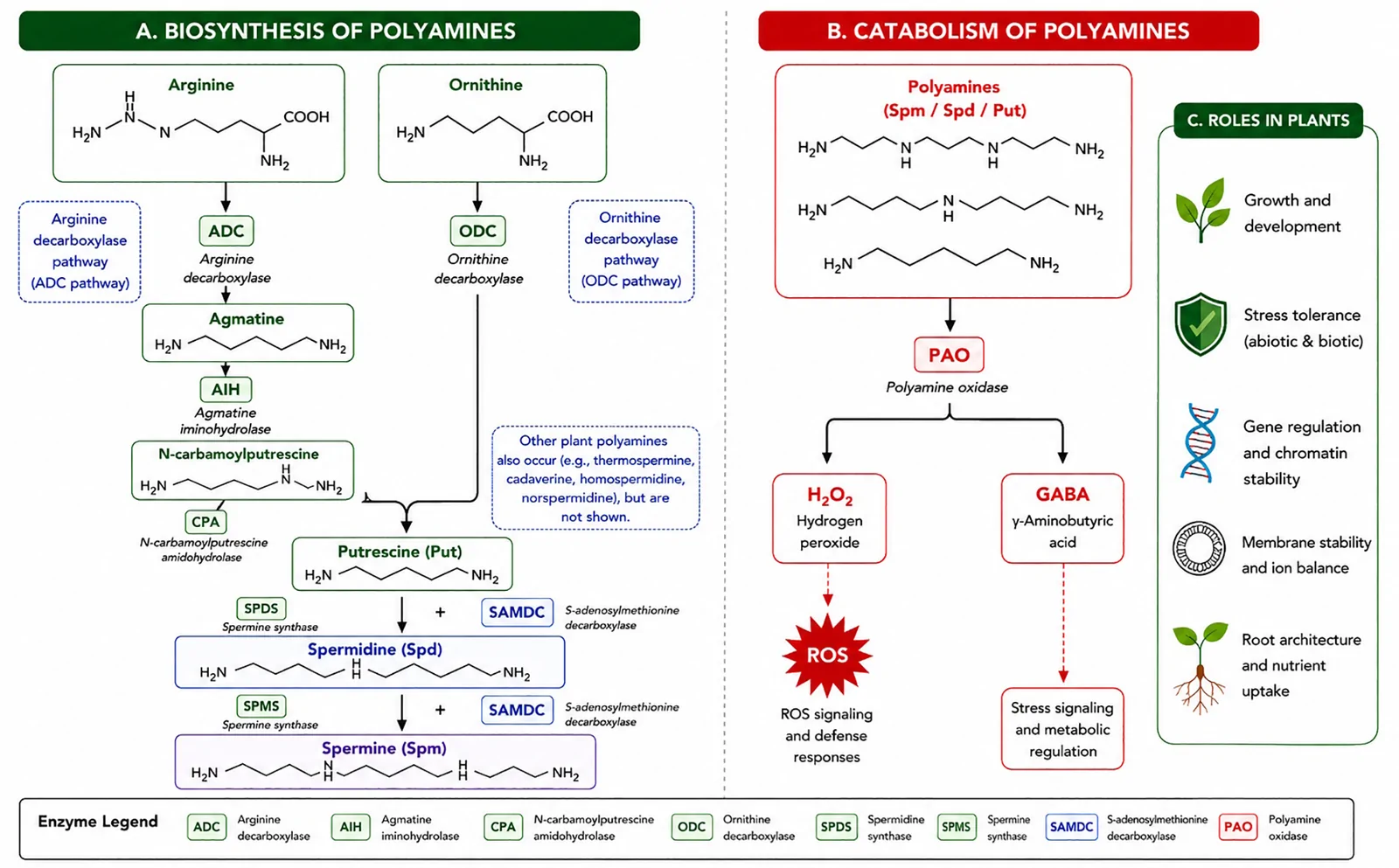
Biosynthesis, catabolism, and major physiological roles of polyamines in plants. Putrescine is synthesized from arginine and ornithine through the arginine decarboxylase (ADC) and ornithine decarboxylase (ODC) pathways, respectively. Putrescine is subsequently converted into spermidine and spermine by the coordinated action of S-adenosylmethionine decarboxylase (SAMDC), spermidine synthase (SPDS), and spermine synthase (SPMS). Polyamine catabolism, mediated by polyamine oxidase (PAO), generates signaling molecules including hydrogen peroxide (H_2_O_2_) and γ-aminobutyric acid (GABA), which participate in stress signaling and metabolic regulation. The figure also summarizes the major physiological functions of PAs in plant growth and development, stress tolerance, gene regulation, membrane stability, and root architecture.

These interconnected biosynthetic and catabolic pathways provide the metabolic foundation for the diverse signaling functions of PAs discussed in the following sections, particularly their roles in regulating plant–microbe interactions, hormonal crosstalk, and stress adaptation.

The intricate pathways of stress hormone signaling, including key players such as auxin, abscisic acid, jasmonic acid, salicylic acid, and gibberellin, exhibit considerable crosstalk with PAs, highlighting the complex interplay between these vital molecules (Seifi and Shelp, 2019; Takahashi, 2020). Recent molecular evidence has shed light on the fact that polyamine signaling plays a crucial role in the crosstalk that is modulated by various biotic or abiotic stresses (Chen et al., 2019). Several catabolic products of PAs have been identified as critical plant stress signals (Liu et al., 2015). The accumulation of such evidence strongly indicates that PAs may have a comprehensive influence over the early signaling events that occur within the plant-response spectrum to various microorganisms and viruses (Seifi and Shelp, 2019). Therefore, understanding how PAs are modulated by factors such as pathogenicity, mutualism, or commensalism is essential and warrants deeper investigation. PAs can be classified as aliphatic nitrogenous organic compounds characterized by the presence of two or more amino groups. These compounds are ubiquitous in nature and are highly charged at physiological pH levels, attributes that favor their direct interaction with essential cellular components, which include nucleic acids and phospholipids (Minocha et al., 2014). As a result, PAs play significant roles in the metabolism of biological macromolecules, their transport, conformation adjustments, and the regulation of their expression levels (Chen et al., 2019). The involvement of PAs extends to a wide range of biological activities, which encompass processes like cell division, cell growth, the differentiation of cells, the development of various organs, and the aging process of organs, known as senescence (Takahashi, 2020). When discussing abiotic stress, PAs are commonly associated with various forms of stress tolerance. These include drought tolerance, tolerance to high salinity conditions, temperature stress tolerance (both high and low), as well as resistance to the detrimental effects of heavy metal toxicity (Liu et al., 2015; Chen et al., 2019). Generally, studies have shown that the content of PAs in plants correlates positively with their overall degree of tolerance to salt and drought conditions (Minocha et al., 2014). Molecular evidence further indicates that PAs act as upstream signaling molecules, playing a direct role in plant resistance mechanisms against both abiotic and biotic stresses (Seifi and Shelp, 2019). The major PAs identified in plants, along with their biosynthetic origins and functional roles, are summarized in **Table 1**. Through their actions, PAs provide plants with indirect tolerance to biotic stress while also influencing the selection of the appropriate resistance pathway (Seifi and Shelp, 2019). Various components related to polyamine biosynthesis, catabolism, signaling, and transport have been identified across different plant species and patterns, yet the evolutionary trajectories of these processes remain largely unknown (Chen et al., 2019). It is also important to note that the role of PAs in participating in the defense spectrum against environmental stimuli, alongside interactions with pathogens, endophytes, and rhizobia, against conditions such as salinity, drought, heavy metals, and temperature fluctuations remains significantly underreported in current literature. As such, further research is essential to fully elucidate the comprehensive roles that PAs play in plant stress responses (Liu et al., 2015; Seifi and Shelp, 2019).

**Table 1:** Major polyamines in plants, their biosynthetic pathways, and principal physiological functions.

Polyamine	Chemical nature	Biosynthetic precursors	Key enzymes	Major biological functions	Role in stress responses	Key References
Putrescine (Put)	Diamine	Arginine/Ornithine	Arginine pathway: Arginine decarboxylase (ADC), Agmatine iminohydrolase (AIH), *N*-carbamoylputrescine amidohydrolase (CPA); Ornithine pathway: Ornithine decarboxylase (ODC)	Cell division, root growth, early development	Early stress signaling, osmotic adjustment	Liu et al., 2015; Chen et al., 2019; Takahashi and Kakehi, 2010
Spermidine (Spd)	Polyamine (triamine)	Putrescine	S-adenosylmethionine decarboxylase (SAMDC), Spermidine synthase (SPDS)	DNA stabilization, protein synthesis, gene regulation	Drought and salinity tolerance, ROS modulation	Seifi and Shelp, 2019; Takahashi, 2020
Spermine (Spm)	Polyamine (tetraamine)	Spermidine	Spermine synthase (SPMS)	Membrane stability, enzyme regulation, signaling	Heat tolerance, pathogen resistance, oxidative stress protection	Minocha et al., 2014; Chen et al., 2019

## 3 Polyamine responses in beneficial and pathogenic plant–microbe interactions

The particular forms that plant–microbe interactions take reflect the intricate and complex evolutionary histories of the various partners involved (Compant et al., 2019; Trivedi et al., 2020). In most plant species, the vast array of root-associated microorganisms can be broadly classified into three main categories: mutualistic, parasitic, and saprophytic (Vandenkoornhuyse et al., 2015). These categories, however, extend beyond just the roots, encompassing organisms that colonize other plant organs (Compant et al., 2019). Members of each of these groups actively intervene in a variety of plant physiological processes and significantly influence plant productivity, growth, and overall fitness—either positively or negatively—dependent on the specific genetic setups or biological types present in the plants (Vandenkoornhuyse et al., 2015; Trivedi et al., 2020). Research has shown that polyamine concentration rises considerably in plants during mutualistic, biotrophic, necrotrophic, and general-pathogen inoculation of plant tissues (Valdés-Santiago and Ruiz-Herrera, 2014; Seifi and Shelp, 2019). The differential responses of PAs across various plant–microbe interaction types are summarized in **Table 2**. Polyamine accumulation patterns differ markedly depending on the nature of plant–microbe interactions. Beneficial associations typically promote the accumulation of higher PAs such as spermidine and spermine, which are associated with growth promotion and stress resilience. In contrast, pathogenic interactions often induce elevated levels of putrescine, which is closely linked to defense signaling and ROS production. These contrasting patterns highlight the role of PAs as key modulators in determining the outcome of plant–microbe interactions.

**Table 2:** Differential modulation of polyamine levels in plants during various plant–microbe interactions. Polyamine responses vary depending on the type of microbial association, contributing to growth promotion, stress tolerance, or activation of defense mechanisms.

Interaction type	Microbial group	Example organisms	Predominant source of polyamines	Polyamine response	Physiological outcome in plant	Key references
Mutualistic	AMF, Rhizobia, PGPR	*Glomus* spp., *Rhizobium* spp., *Bacillus* spp.	Plant + beneficial microorganisms	↑ Spermidine, ↑ Spermine	Enhanced growth, nutrient uptake, improved symbiosis, stress tolerance	Compant et al., 2019; Begum et al., 2019; Trivedi et al., 2020; Dunn and Becerra-Rivera, 2023
Endophytic	Non-pathogenic fungi/bacteria	*Trichoderma* spp., *Enterobacter* spp.	Plant + endophytic microorganisms	Moderate ↑ Polyamines	Defense priming, improved root development, enhanced stress adaptation	Woo et al., 2014; Compant et al., 2019; Seifi and Shelp, 2019; Dunn and Becerra-Rivera, 2023
Pathogenic (Biotrophic)	Fungi, bacteria	*Xanthomonas* spp., rust fungi	Predominantly plant (defense response); pathogen-derived polyamines may also contribute	↑ Putrescine	Activation of defense signaling, ROS production	Jiménez-Bremont et al., 2014; Seifi and Shelp, 2019
Pathogenic (Necrotrophic)	Fungi	*Fusarium* spp., *Botrytis* spp.	Predominantly plant; pathogen polyamines may influence virulence	Strong ↑ Putrescine, altered Spd/Spm ratio	Programmed cell death, disease progression	Jiménez-Bremont et al., 2014; Valdés-Santiago and Ruiz-Herrera, 2014; Seifi and Shelp, 2019
Neutral/Commensal	Soil microbiota	Diverse rhizosphere microbes	Plant + rhizosphere microbial community	Stable or slight changes	Homeostasis maintenance	Vandenkoornhuyse et al., 2015; Trivedi et al., 2020

↑ means increasing.

For instance, in the case of a specific biotrophic fungus that thrives in temperate climates, a remarkably similar response occurs during saprophytic infection, which is observed even after just the first exposure to hypoxic conditions (Valdés-Santiago and Ruiz-Herrera, 2014). This indicates that environmental factors can significantly affect plant responses to microbial interactions (Trivedi et al., 2020). In contrast, the application of certain plant growth-promoting bacteria (PGPB), such as *Pseudomonas fluorescens*, *Bacillus subtilis*, and *Azospirillum brasilense*, which generally do not induce major alterations in root morphology, has been reported to increase polyamine accumulation in host plants (Seifi and Shelp, 2019). Such observations suggest that polyamine-mediated signaling may activate distinct regulatory pathways depending on the interacting microorganism. In beneficial plant–microbe interactions, these effects may result from multiple sources of PAs. Endogenous PAs synthesized by the host plant contribute to the regulation of root development, cellular signaling, and symbiotic compatibility, whereas several beneficial microorganisms, including arbuscular mycorrhizal fungi, rhizobia, plant growth-promoting rhizobacteria (PGPR), and endophytes, also produce PAs that facilitate microbial colonization, biofilm formation, nutrient exchange, and establishment of mutualistic associations. In addition, exogenous application of PAs has been shown to enhance symbiotic efficiency and improve plant adaptation under stressful environmental conditions. Together, these findings indicate that plant–microbe interactions are regulated through the combined contribution of endogenous, microbial-derived, and externally applied PAs rather than by a single source of these metabolites (Jiménez-Bremont et al., 2014; Seifi and Shelp, 2019; Dunn and Becerra-Rivera, 2023). This functional divergence highlights the dynamic role of PAs as signaling molecules that regulate the outcome of plant–microbe interactions according to the lifestyle of the microbial partner (Compant et al., 2019; Trivedi et al., 2020; Lino-Neto and Baptista, 2022).

## 4 Mechanisms of polyamine-mediated crosstalk in plant–microbe interactions

PAs are low-molecular-weight aliphatic compounds with a unique nitrogenous structure. In plants, they comprise three basic compounds: putrescine (1,4-diaminobutane), spermidine (N-(3-aminopropyl) putrescine), and spermine (N,N′-bis(3-aminopropyl) putrescine) (Chen et al., 2019; Takahashi, 2020). PAs in plants evolve from the amino acid ornithine and precursor 1,1-aminopropane (Liu et al., 2015). Different PAs contribute to a variety of physiological activities in higher plants, such as cell division, cell elongation, seed germination, stomatal opening, formation of conductive elements, biomass production, detachment of abscission layer, senescence, and the responses to biotic and abiotic stress (Minocha et al., 2014; Chen et al., 2019). The general biosynthesis, accumulation, and physiological roles of PAs in plants are well documented (Liu et al., 2015). The mineral nutrients in the soil are taken up, transported, distributed, and accumulated in the tissues of plants and are necessary, but insufficient, to maintain the healthy growth of plants. It is probable that microbial communities that developed billions of years ago on earth control these processes by providing chemical signals (Trivedi et al., 2020). Beneficial interactions of mycorrhizae, Rhizobia, and certain soil bacteria with plants enhance the physiological attributes of the hosting plants, and specific signal exchanges between plants and microbes are identified (Vandenkoornhuyse et al., 2015; Compant et al., 2019). The mineral ions taken up from the soil are also modified by microorganisms that enrich the common soil borne microbes detected in plants (Jiménez-Bremont et al., 2014). Colonization of the roots, nodulation of leguminous crops, ectomycorrhizal and arbuscular mycorrhizal formation, interactions with nematodes and earthworms, and infection with harmful microbes could alter the physiological condition of plants at the very first step (Vandenkoornhuyse et al., 2015). Commonly, signal exchanges between the microbes and plants are recognized by root exudates, chemical signals released from roots, and the presence and composition of the soil borne microbes, and the surrounding environmental stimuli determine if the microbial interactions would turn to the beneficial or harmful events (Compant et al., 2019; Trivedi et al., 2020). Pathogenic microbes such as bacteria, fungi, and viruses infection through specific signaling routes, active defense and pre-defensive signals are triggered to defend against a number of harmful microbes, and the composition of biochemical signal molecules changes drastically (Minocha et al., 2014; Seifi and Shelp, 2019). The mechanistic processes through which PAs regulate plant–microbe interactions are illustrated in **Figure 2**. PAs function as central regulators that integrate biosynthesis, catabolism, rhizosphere signaling, and communication between plants and microorganisms. In addition to coordinating crosstalk with ROS, nitric oxide (NO), and phytohormone signaling pathways, PAs modulate the activities of beneficial microorganisms, including plant growth-promoting rhizobacteria (PGPR), arbuscular mycorrhizal fungi (AMF), and endophytes, while contributing to defense against pathogenic microbes. The figure further illustrates how polyamine-mediated signaling influences microbial community dynamics, gene expression, nutrient acquisition, redox homeostasis, plant growth, stress tolerance, and disease resistance through interconnected physiological and molecular pathways (Mittler, 2017; Waszczak et al., 2018; Hasanuzzaman et al., 2020; Hasan et al., 2021).

**Figure 2:**
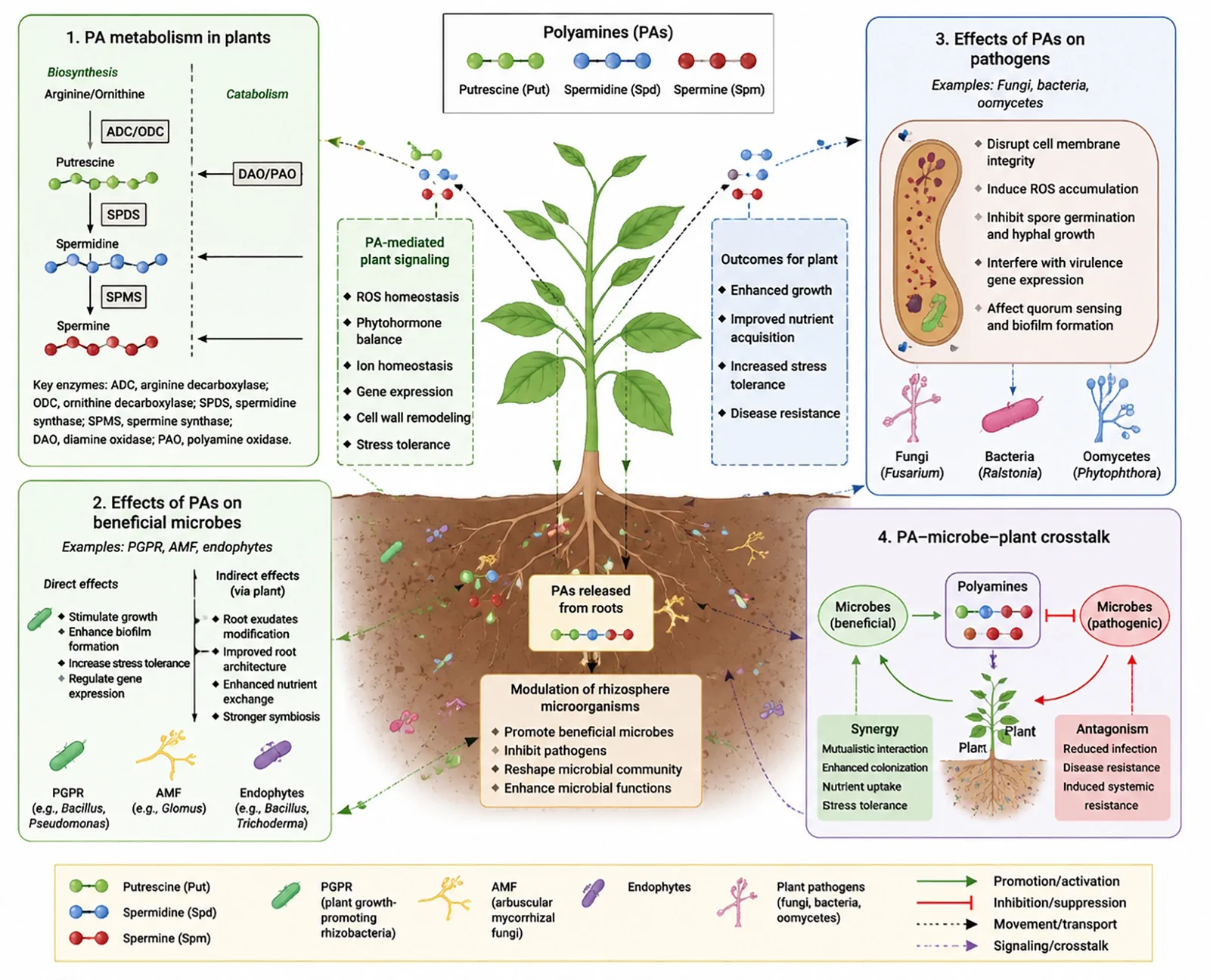
Mechanistic model of polyamine-mediated regulation of plant–microbe interactions. Polyamines (putrescine, spermidine, and spermine) are synthesized through the arginine/ornithine pathway and participate in dynamic signaling processes that regulate communication between plants and microorganisms. They influence beneficial microbes, including plant growth-promoting rhizobacteria (PGPR), arbuscular mycorrhizal fungi (AMF), and endophytes, while suppressing pathogen development by modulating ROS homeostasis, phytohormone signaling, ion balance, gene expression, and defense responses. PAs released into the rhizosphere further shape microbial community composition and strengthen plant–microbe crosstalk, ultimately promoting nutrient acquisition, plant growth, stress tolerance, and disease resistance.

### 4.1 Signal transduction and gene regulation

PAs are known to play central roles in various plant–microbe interactions, highlighting their importance in the ecological dynamics between plants and microorganisms (Jiménez-Bremont et al., 2014; Seifi and Shelp, 2019). Interactive microbes have been shown to induce biosynthesis of PAs, while simultaneously, these PAs significantly shape the responses of the host plants to their environments (Liu et al., 2015; Chen et al., 2019). The functioning of PAs occurs through intricate crosstalk with various signaling molecules and transcriptional regulators, allowing them to effectively transduce both exogenous and endogenous signals within the plant systems (Seifi and Shelp, 2019). Notably, there are four primary pathways that synthesize the predominant PAs: putrescine, spermidine, and spermine (Chen et al., 2019; Takahashi, 2020). Meanwhile, various catabolic activities give rise to crucial signaling molecules such as reactive aldehydes and γ-aminobutyric acid (GABA), which play important roles in stress adaptation, metabolic regulation, and plant–microbe signaling (Liu et al., 2015; Seifi and Shelp, 2019; Sobieszczuk-Nowicka and Legocka, 2014). The signals derived from PAs have a wide-ranging influence on the levels of ROS, the balance of redox processes, and the overall antioxidant systems that are activated during diverse microbial encounters (Seifi and Shelp, 2019; Chen et al., 2019). These alterations in cellular processes contribute to enhanced drought tolerance and improved disease resistance in polyamine-responsive germplasm, significantly impacting agricultural practices (Liu et al., 2015). Within the context of plant responses, the pathways involving auxin, abscisic acid, ethylene, jasmonic acid, and salicylic acid interact dynamically with PAs, revealing their complexity in biotic stress situations (Seifi and Shelp, 2019; Takahashi, 2020). PAs not only modulate the responses to beneficial microbes which can enhance growth and development but also play a critical role in the responses to pathogenic microbes (Jiménez-Bremont et al., 2014; Liu et al., 2015). In beneficial associations, these compounds can significantly affect developmental priming and are integral to the cumulative transduction processes that follow (Seifi and Shelp, 2019). Conversely, during instances of infection, PAs have been implicated in conferring resistance against the pathogens (Liu et al., 2015; Chen et al., 2019). Although the three major PAs frequently act in a coordinated manner, increasing evidence indicates that they also perform partially distinct physiological functions. Putrescine is primarily associated with early stress perception, osmotic adjustment, and abscisic acid (ABA)-mediated signaling during drought and salinity stress, particularly in *Arabidopsis thaliana*, rice (*Oryza sativa*), and tomato (*Solanum lycopersicum*). Spermidine has been more closely linked to chromatin stabilization, regulation of stress-responsive gene expression, antioxidant activation, and the establishment of beneficial interactions with plant growth-promoting rhizobacteria (PGPR) and arbuscular mycorrhizal fungi (AMF). In contrast, spermine is frequently associated with membrane stabilization, salicylic acid- and jasmonic acid-dependent defense signaling, and enhanced resistance against pathogenic fungi and bacteria. Nevertheless, these functional distinctions are not universal and remain dependent on plant species, developmental stage, microbial lifestyle, and environmental conditions (Jiménez-Bremont et al., 2014; Seifi and Shelp, 2019; Takahashi, 2020). As a result, experimental strategies geared toward exploiting these insights could offer innovative avenues for enhancing stress resilience, promoting effective biocontrol methods, and facilitating the management of soil microbiomes for improved crop health and productivity (Jiménez-Bremont et al., 2014; Liu et al., 2015; Seifi and Shelp, 2019; Trivedi et al., 2020).

### 4.2 Modulation of ROS and cellular redox homeostasis

ROS are critically important signaling molecules in plants that play a pivotal role in shaping their responses to a wide array of environmental stimuli, including a range of both biotic and abiotic challenges (Mittler, 2017; Waszczak et al., 2018). Under conditions of stress, ROS levels can rapidly increase, leading to alterations in redox homeostasis that have the potential to cause deleterious effects on a variety of cellular molecules such as DNA, RNA, proteins, and lipids (Mittler, 2017). When ROS levels become elevated, they also contribute significantly to the establishment of programmed cell death (PCD) and activate various defense networks within the plant (Waszczak et al., 2018). PAs, which are polycationic compounds, are found in both plants and microbes, and they exert important physiological roles within both kingdoms of life (Jiménez-Bremont et al., 2014; Chen et al., 2019). Microbial species produce PAs that can efficiently penetrate the plant cell wall, subsequently eliciting a diverse range of plant responses (Jiménez-Bremont et al., 2014). The introduction of exogenous PAs has been shown to influence ROS production and to modulate numerous signaling pathways, along with various plant responses to both biotic and abiotic stressors (Kusano and Suzuki, 2015; Seifi and Shelp, 2019). The modulation of ROS is a crucial component of stress bioengineering initiatives, which aim to design and develop crops capable of achieving higher biomass, or tolerating various abiotic and biotic stresses when exposed to natural field conditions (Mittler, 2017). Crosstalk between ROS and individual PAs has been documented in several plant species exposed to both abiotic and biotic stresses. Putrescine primarily contributes to early ROS signaling during stress perception and defense activation, whereas spermidine and spermine are more frequently associated with activation of antioxidant enzymes, maintenance of redox homeostasis, and protection against oxidative damage. For example, increased putrescine accumulation has been reported during pathogen challenge in *Arabidopsis thaliana*, while spermidine- and spermine-mediated antioxidant responses have been demonstrated in crops such as wheat, rice, and tomato subjected to drought, salinity, and temperature stress (Kusano and Suzuki, 2015; Seifi and Shelp, 2019; Hasanuzzaman et al., 2020). These observations indicate that although the three major PAs cooperate in regulating ROS homeostasis, they often contribute to different stages and components of the stress response depending on the biological context.

### 4.3 Polyamine catabolism and production of signaling molecules

PAs are organic compounds characterized by two or more amino groups. In plants, they are categorized into three classes: (1) aliphatic compounds comprising diamines such as putrescine (Put) and PAs such as spermine (Spm) and spermidine (Spd), (2) aromatic PAs like phenol-2,4-diamine (caffein), and (3) PAs with both aliphatic and aromatic chemical structures such as N,N-bis (3-aminopropyl) m-aminobenzene sulfonamide (BGC) (Chen et al., 2019; Takahashi, 2020). Since increasing attention has been paid to PAs over the past two decades, several important roles of these molecules in biotic and abiotic stress tolerance, plant–pathogen interactions, and disease resistance have emerged (Minocha et al., 2014; Seifi and Shelp, 2019) when compared to the much longer established characterization of non-PA signaling molecules in regulating plant–microbe relationships and host immunity. Different microorganisms elicit diverse responses from plants. Microorganisms can form beneficial interactions with plants like symbiosis (establishment of N_2_-fixing root nodules by diazotrophic bacteria), mycorrhiza with mycorrhizal fungi, endophytism (formation of fungal endophytes), and associative diazotrophy at soil-root interfaces (Vandenkoornhuyse et al., 2015; Trivedi et al., 2020). Under specific circumstances, however, both pathogens and beneficial symbionts may engage with similar signaling molecules and parallel pathways (Jiménez-Bremont et al., 2014; Seifi and Shelp, 2019). Higher organisms, plants included, exploit complex mechanisms of crosstalk between signaling pathways involved in the receipt, amplification, and transduction of extracellular biotic and abiotic stimuli (Mittler, 2017; Waszczak et al., 2018). The existence of such larger, integrated networks obliges consideration of plant responses to complex mixtures of different biotic and abiotic stresses occurring simultaneously (Trivedi et al., 2020). PAs have emerged as universal signaling and regulatory molecules capable of counteracting the deleterious effects of a truly impressive array of noxious parameters while preserving the health of myriad plants and acting also in the modeling of specific multi-signaling influences upon microbial (bio)systems (Seifi and Shelp, 2019; Chen et al., 2019). In plants, signaling of PAs appears tightly linked to signaling by the indole-3-acetic acid molecule (IAAs), one of the most studied plant hormones (Seifi and Shelp, 2019; Takahashi, 2020).

### 4.4 Hormonal interplay and stress signaling networks

PAs play an essential role in regulating the complex interplay among plant hormones, including auxins, abscisic acid, ethylene, jasmonic acid, salicylic acid, and brassinosteroids (Seifi and Shelp, 2019; Chen et al., 2019; Menéndez et al., 2019). These hormones are deeply involved in the response mechanisms to a wide array of biotic, such as pests and pathogens, and abiotic stress signals, which include environmental factors like drought, salinity, and temperature extremes (Minocha et al., 2014; Verma et al., 2016). This intricate regulation significantly modulates gene expression and influences the complex signal transduction pathways that are absolutely vital for the adaptive responses of plants (Chen et al., 2019). For instance, PAs can enhance the biosynthesis of auxins during beneficial interactions with other organisms, thereby promoting overall growth and development in favorable conditions (Seifi and Shelp, 2019). On the other side, the catabolism of PAs, along with the synthesis and production of various associated molecules, represents alternative yet crucial metabolic routes that allow plants to fine-tune their responses to diverse biotic stimuli (Liu et al., 2015; Seifi and Shelp, 2019). This fine-tuning effectively improves their resilience against harmful pests and devastating diseases (Verma et al., 2016). When considering the context of abiotic stressors, PAs synergistically work in concert with beneficial microbes to confer much-enhanced tolerance to a variety of challenging environmental stressors, such as extreme drought conditions, high salinity, soaring heat, and severe cold (Liu et al., 2015; Chen et al., 2019). This cooperation is of paramount importance in regulating hormonal balance and activating highly specific stress-related networks that enable plants to effectively cope with these adverse environmental conditions (Seifi and Shelp, 2019). Overall, the dynamic interaction between PAs and various hormonal pathways is fundamental for optimizing plant performance under both stressful and favorable conditions, ensuring the survival and growth of plants in a constantly changing environment (Minocha et al., 2014; Verma et al., 2016; Chen et al., 2019). Collectively, these findings demonstrate that PAs function as central regulators of plant–microbe interactions by integrating biosynthetic and catabolic pathways, redox homeostasis, hormonal signaling, and rhizosphere microbial processes. However, available evidence also suggests functional specialization among the major plant PAs. Putrescine is predominantly associated with early stress perception, osmotic adjustment, and ABA-dependent responses; spermidine contributes mainly to antioxidant regulation, transcriptional control, and beneficial plant–microbe interactions; whereas spermine is more frequently linked to membrane stabilization, salicylic acid- and jasmonic acid-mediated defense signaling, and long-term adaptation to biotic and abiotic stresses. These functional differences have been reported in several model and crop species, including *Arabidopsis thaliana*, rice (*Oryza sativa*), wheat (*Triticum aestivum*), and tomato (*Solanum lycopersicum*), although their relative contributions remain dependent on plant species, developmental stage, environmental conditions, and the lifestyle of the interacting microorganism. Further comparative studies are therefore required to define species-specific regulatory mechanisms and facilitate the application of polyamine-mediated strategies in sustainable crop production.

## 5 Recent advances in polyamine-mediated abiotic and biotic stress tolerance

Recent studies have established PAs as central regulators of plant stress adaptation through their ability to integrate osmotic adjustment, ion homeostasis, ROS detoxification, hormonal signaling, and plant–microbe communication (Chen et al., 2019; Seifi and Shelp, 2019; Menéndez et al., 2019). Rather than functioning solely as stress-induced metabolites, putrescine, spermidine, and spermine actively participate in complex signaling networks that coordinate plant responses to drought, salinity, temperature extremes, heavy metal toxicity, and pathogen attack (Liu et al., 2015; Hasanuzzaman et al., 2020). Increasing evidence also indicates that polyamine-mediated signaling is closely linked with beneficial microorganisms, including plant growth-promoting rhizobacteria (PGPR) and arbuscular mycorrhizal fungi (AMF), thereby enhancing plant resilience under adverse environmental conditions. These recent advances highlight the potential of PAs as key regulators of stress adaptation and promising targets for improving crop performance and sustainable agricultural production. The major roles of PAs in mediating plant responses to different abiotic stresses are summarized in **Table 3**.

**Table 3:** Modulation of polyamine levels in plants under major abiotic stresses and their associated physiological and molecular mechanisms contributing to stress tolerance.

Abiotic stress	Dominant polyamine response	Representative plant species	Key mechanisms	Physiological effects	Outcome	Key references
Drought	↑ Putrescine, ↑ Spermine	*Oryza sativa*, *Arabidopsis thaliana*, *Solanum lycopersicum*	Stomatal regulation, osmotic adjustment, proline accumulation, ROS scavenging	Reduced water loss, improved water-use efficiency, enhanced antioxidant defense	Enhanced drought tolerance	Liu et al., 2015; Chen et al., 2019; Hasan et al., 2021; Upadhyay et al., 2020
Salinity	↑ Spermidine, ↑ Putrescine	*Triticum aestivum*, *Oryza sativa*, *Arabidopsis thaliana*	Na^+^/K^+^ homeostasis, membrane stabilization, antioxidant activation, proline accumulation	Reduced ionic toxicity, improved cellular integrity, enhanced osmotic balance	Salt stress tolerance	Gill and Tuteja, 2010; Liu et al., 2015; Chen et al., 2019; Upadhyay et al., 2020
Heat Stress	↑ Spermine	*Arabidopsis thaliana*, wheat, tomato	Membrane protection, protein stabilization, antioxidant activation	Reduced protein denaturation, improved photosynthetic performance	Enhanced thermotolerance	Chen et al., 2019; Upadhyay et al., 2020
Cold Stress	↑ Putrescine	*Arabidopsis thaliana*, rice	ROS detoxification, stabilization of cellular structures, membrane protection	Reduced oxidative damage, improved membrane integrity	Cold tolerance	Alet et al., 2011; Chen et al., 2019; Upadhyay et al., 2020
Heavy Metal Stress	↑ Putrescine, ↑ Spermidine	*Brassica juncea*, *Arabidopsis thaliana*	Metal chelation, antioxidant defense, detoxification pathways, maintenance of redox homeostasis	Reduced metal toxicity, enhanced cellular protection	Improved survival under metal toxicity	Minocha et al., 2014; Liu et al., 2015; Chen et al., 2019

↑ means increasing.

Polyamine accumulation under abiotic stress conditions follows stress-specific patterns that contribute to adaptive responses in plants (Gill and Tuteja, 2010). For instance, drought and salinity stresses are associated with increased levels of putrescine and spermidine, facilitating osmotic adjustment and ion homeostasis (Ahmad et al., 2010; Hasanuzzaman et al., 2020). In contrast, spermine accumulation is more closely linked to heat stress tolerance through membrane stabilization and protein protection. These differential responses highlight the role of PAs as key modulators of stress-specific physiological processes and underline their importance in enhancing plant resilience under adverse environmental conditions. The roles of PAs in mediating plant responses to both abiotic and biotic stresses are illustrated in **Figure 3**.

**Figure 3:**
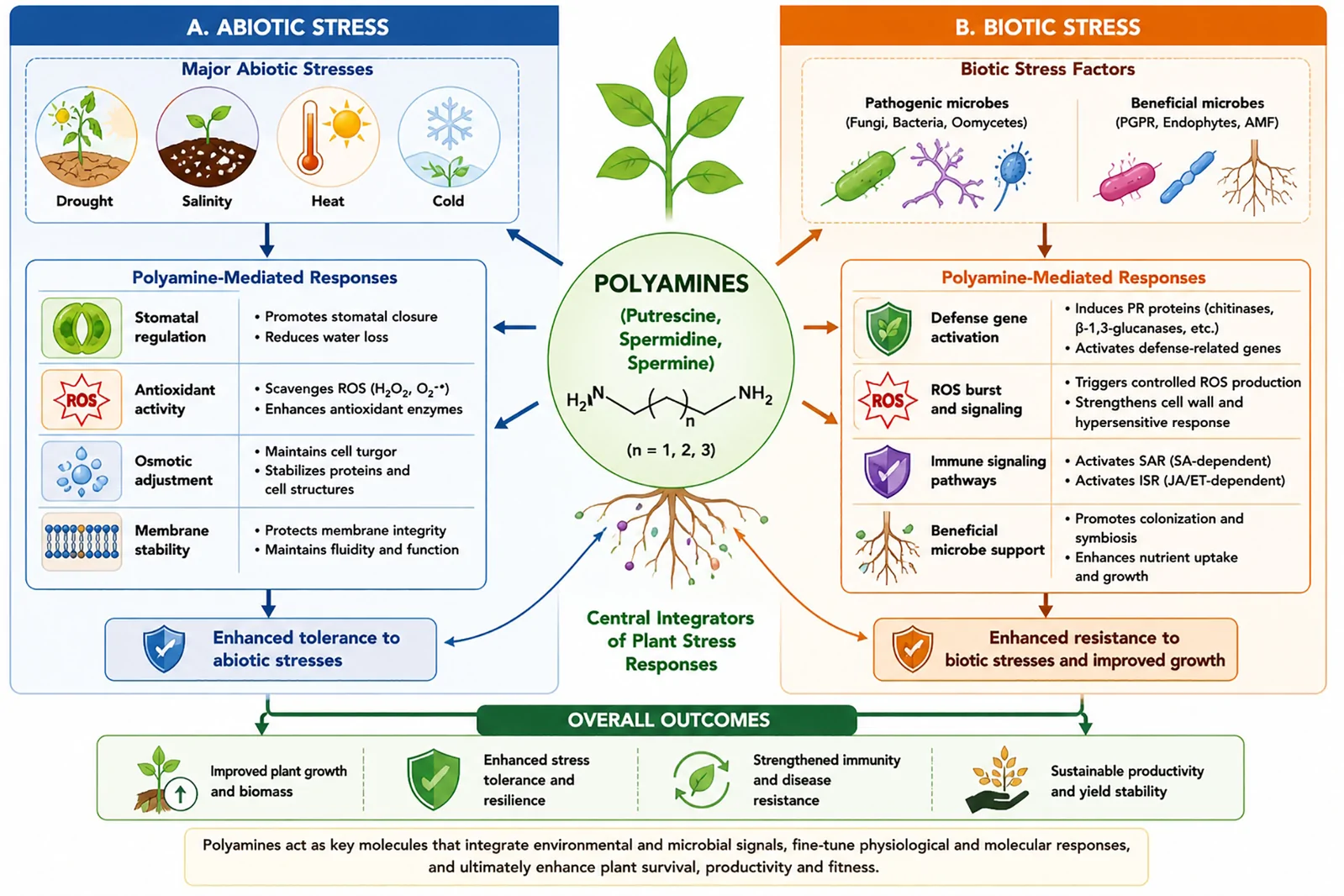
Roles of polyamines in plant responses to abiotic and biotic stresses. Under abiotic stress conditions such as drought, salinity, heat, and cold, PAs regulate stomatal closure, antioxidant activity, and membrane stability. In biotic stress conditions, PAs contribute to defense responses through activation of pathogenesis-related proteins, ROS production, and modulation of plant immunity. These combined mechanisms enhance plant resilience and stress tolerance.

PAs play a dual role in mediating plant responses to both abiotic and biotic stresses by integrating physiological and molecular mechanisms. Under abiotic stress conditions, they regulate stomatal behavior, antioxidant defense systems, and membrane stability, thereby enhancing plant tolerance to environmental constraints. In contrast, during biotic stress, PAs contribute to the activation of defense-related pathways, including ROS production and pathogenesis-related gene expression. This dual functionality highlights their importance as central regulators in plant stress biology and supports their potential application in improving crop resilience. When faced with conditions of drought and salinity, PAs present in these systems promote the process of stomatal closure and boost antioxidant activity, which collectively enhance the resilience of plants to various forms of abiotic stress. Additionally, PAs are instrumental in counteracting the detrimental effects of both heat and cold stress in the roots and leaves of plants, thereby improving cell membrane stability and effectively reducing electrolyte leakage. Furthermore, these valuable compounds show effectiveness in counteracting seedling blight caused by harmful *Fusarium* species and significantly enhance the growth of seedlings after transplanting, ensuring greater survival rates and healthier development (Kissoudis et al., 2014; Liu et al., 2015). Although considerable progress has been made in elucidating the role of PAs in plant–microbe interactions, accumulating evidence indicates that their regulatory functions are highly context dependent rather than universally conserved. The magnitude and direction of polyamine-mediated responses vary according to plant species, developmental stage, microbial lifestyle, and environmental conditions (Seifi and Shelp, 2019; Takahashi, 2020; Kusano and Suzuki, 2015). For example, recent studies have demonstrated extensive crosstalk between PAs and brassinosteroid signaling, where brassinosteroids modulate polyamine biosynthesis while PAs, in turn, influence BR-dependent growth and stress-responsive pathways (Kusano and Suzuki, 2015). Likewise, the close metabolic and signaling relationship between PAs and γ-aminobutyric acid (GABA), mediated through polyamine catabolism, provides an additional regulatory layer integrating carbon and nitrogen metabolism, redox homeostasis, and stress adaptation (Seifi and Shelp, 2019). These interactions emphasize that PAs should be regarded not as isolated signaling molecules but as components of an interconnected regulatory network coordinating hormonal, metabolic, and microbial signals (Jiménez-Bremont et al., 2014; Kusano and Suzuki, 2015). Future studies should therefore focus on dissecting these species-specific regulatory mechanisms under realistic environmental conditions to facilitate the translation of laboratory findings into agricultural applications.

### 5.1 Drought and salinity stress responses

Accumulating evidence indicates that PAs play a significant role in plant responses to drought and salinity (Liu et al., 2015; Chen et al., 2019). High salinity and drought conditions increase osmotic stress, causing the loss of water and inhibition of photosynthesis, accumulation of ROS, and oxidative damage (Mittler, 2017; Waszczak et al., 2018). Substantial alterations in plant endogenous polyamine concentrations are often observed under these abiotic stresses (Liu et al., 2015). Drought conditions elevate putrescine and spermine, while salinity increases spermidine, putrescine, and spermine concentrations in several crop species (Minocha et al., 2014; Chen et al., 2019). PAs are a class of ubiquitous aliphatic organic compounds that are essential for growth and development in all organisms and have been implicated as signaling molecules in both plants and microbes (Jiménez-Bremont et al., 2014; Seifi and Shelp, 2019). Cyclin-dependent kinase inhibitors (CKIs) have emerged as central regulators of the endocycle that regulate the entry into the next cycle; in silkworm PAs modulate the expression of four target genes of ecdysone signaling (Minocha et al., 2014). Microalgae produce PAs from carbohydrate and nitrogen sources under diverse environmental conditions, including nitrogen starvation, salt stress, high CO_2_ concentration, and illumination (Chen et al., 2019). High salinity and drought conditions increase osmotic stress, causing loss water and inhibition of photosynthesis, accumulation of ROS and oxidative damage (Mittler, 2017; Waszczak et al., 2018). Salinity induces significant alterations in plant endogenous polyamine concentrations and both abiotic stresses also affect microbiome structure (Liu et al., 2015; Trivedi et al., 2020). Endophytic microbes and plant growth-promoting microbes have been shown to alter stress response and may mediate crosstalk between plants and PAs (Compant et al., 2019; Trivedi et al., 2020).

Recent experimental studies have provided more detailed insight into the physiological functions of individual PAs during drought and salinity stress. In rice (*Oryza sativa*), exogenous spermidine (0.5–1.0 mM) significantly improved drought tolerance by enhancing antioxidant enzyme activities, maintaining chlorophyll content, and reducing membrane lipid peroxidation. In wheat (*Triticum aestivum*), spermine application (0.1–0.5 mM) alleviated oxidative damage under salinity stress (100–150 mM NaCl) through improved membrane stability, increased activities of superoxide dismutase (SOD), catalase (CAT), and peroxidase (POD), and reduced electrolyte leakage. Conversely, putrescine accumulates rapidly during the early stages of osmotic stress and contributes to osmotic adjustment through the promotion of compatible solutes, particularly proline, together with soluble sugars, thereby helping maintain cellular water status during drought and salt stress. These findings indicate that although the three major PAs act cooperatively, they contribute to different physiological components of stress adaptation, with putrescine functioning predominantly during early stress perception and osmotic adjustment, whereas spermidine and spermine mainly enhance antioxidant defense, membrane protection, and long-term stress acclimation (Hasanuzzaman et al., 2020; Upadhyay et al., 2020; Hasan et al., 2021; Dunn and Becerra-Rivera, 2023). Beyond endogenous regulation, exogenous application of PAs through seed priming and foliar treatments has also been shown to improve drought and salinity tolerance by enhancing antioxidant activity, maintaining membrane integrity, and promoting osmotic adjustment. Such treatments complement endogenous PA metabolism and represent practical approaches for improving crop resilience under water-deficit and saline conditions (Chen et al., 2019; Seifi and Shelp, 2019).

### 5.2 Heat and cold stress adaptations

Temperature extremes severely affect plant growth, development, and productivity by disrupting membrane integrity, protein stability, photosynthesis, and cellular redox homeostasis (Mittler, 2017; Waszczak et al., 2018). To cope with these adverse conditions, plants activate complex physiological and molecular responses involving stress-responsive genes, antioxidant systems, and signaling molecules that collectively enhance cellular protection and survival (Mittler, 2017). PAs have emerged as important components of these adaptive responses by stabilizing cellular membranes, protecting proteins, regulating antioxidant defenses, and maintaining cellular homeostasis under both heat and cold stress (Liu et al., 2015; Chen et al., 2019). Furthermore, increasing evidence suggests that beneficial microorganisms can influence polyamine metabolism and thereby contribute to improved plant tolerance to temperature stress through coordinated physiological and biochemical responses (Seifi and Shelp, 2019). Recent studies have further demonstrated that heat and cold stresses differentially regulate polyamine biosynthesis and catabolism genes. Stress-specific changes in putrescine, spermidine, and spermine metabolism have been associated with membrane stabilization, antioxidant activation, protection of cellular proteins, and maintenance of photosynthetic efficiency. For example, differential regulation of polyamine metabolic pathways under heat and cold stress has been reported in tomato, indicating that polyamine metabolism is dynamically adjusted according to the nature and intensity of temperature stress rather than being uniformly activated under all environmental conditions (Upadhyay et al., 2020). These findings highlight the potential of manipulating polyamine metabolism, either through exogenous application or in combination with beneficial microorganisms, to improve crop resilience under increasingly variable climatic conditions (Chen et al., 2019; Seifi and Shelp, 2019). Recent advances further suggest that manipulation of PA biosynthetic pathways through breeding and genetic approaches represents a promising strategy for improving crop tolerance to temperature extremes. Enhanced endogenous PA biosynthesis has been associated with improved membrane stability, antioxidant capacity, and protection of photosynthetic machinery under heat and cold stress, providing opportunities for developing climate-resilient crop cultivars (Chen et al., 2019; Begum et al., 2019; Takahashi, 2020).

### 5.3 Disease resistance and seedling establishment

PAs play an important role in enhancing plant resistance to biotic stresses by regulating defense signaling, ROS production, and the expression of disease-resistance-related genes (Jiménez-Bremont et al., 2014; Seifi and Shelp, 2019). During seed germination and early seedling establishment, both endogenous and exogenously applied PAs strengthen plant defenses against major soil-borne pathogens, including *Fusarium* spp., *Rhizoctonia solani*, *Sclerotinia sclerotiorum*, *Pythium* spp., and *Phytophthora* spp., which cause damping-off, root rot, and seedling blight (Agrios, 2012; Jiménez-Bremont et al., 2014). In addition to activating antioxidant systems, PAs stimulate the expression of pathogenesis-related proteins and other defense-associated genes, thereby reducing disease severity and improving seedling establishment under pathogen pressure (Seifi and Shelp, 2019). Recent advances have further demonstrated that polyamine-based seed priming represents a promising strategy for enhancing seed germination, seedling vigor, and disease resistance through the activation of antioxidant metabolism and defense-related signaling pathways (Wojtyla et al., 2024). Moreover, increasing evidence suggests that integrating polyamine application with beneficial microorganisms may further enhance induced resistance and early plant establishment by promoting root development and strengthening plant immune responses (Compant et al., 2019; Trivedi et al., 2020). These findings highlight the potential of polyamine-mediated approaches as sustainable tools for integrated disease management and crop protection.

Overall, recent advances demonstrate that PAs function as central integrators of plant responses to both abiotic and biotic stresses rather than merely acting as stress-induced metabolites. Through their interactions with ROS, phytohormones, antioxidant systems, and beneficial microorganisms, PAs coordinate multiple physiological and molecular processes that enhance plant resilience under adverse environmental conditions. These findings further support the potential application of polyamine-mediated strategies in sustainable crop production. Nevertheless, additional research is still required to elucidate crop-specific regulatory mechanisms, validate their effectiveness under field conditions, and optimize their integration with microbial biostimulants and biological control agents for long-term agricultural applications. Integration of PA-mediated regulation with microbial biocontrol strategies represents an emerging approach for sustainable disease management. Combining PA application or manipulation of PA metabolism with beneficial microorganisms such as PGPR, AMF, and endophytes has been shown to enhance induced resistance, improve root colonization, and strengthen plant immune responses, thereby increasing crop resilience against soil-borne pathogens (Compant et al., 2019; Trivedi et al., 2020).

## 6 Applications in crop protection and agricultural practice

The increasing incidence of plant diseases has stimulated efforts to develop sustainable agricultural systems, including strategies aimed at improving soil microbiota composition to enhance plant stress resilience (Leonardi et al., 2026). In this context, PAs have received considerable attention due to their roles in both plants and associated microorganisms. The influence of PAs on plant–microbe interactions has been previously reviewed, highlighting their importance in modulating plant defense responses and microbial communication (Minocha et al., 2014; Seifi and Shelp, 2019). The need to improve stress resilience in adaptable cropping systems has led to increased interest in polyamine-mediated plant–microbe crosstalk. Previous studies have demonstrated that PAs are involved in signaling pathways activated during plant–pathogen interactions, suggesting their role in coordinating defense responses and regulating microbial colonization processes (Jiménez-Bremont et al., 2014; Liu et al., 2015). Exogenous application of PAs, including seed treatments and foliar sprays, has been reported to enhance plant resistance against major soilborne pathogens such as *Phytophthora* spp. and *Fusarium* spp. (Wojtyla et al., 2024). These effects are often associated with modulation of amino acid metabolism, proton balance, and the production of γ-aminobutyric acid (GABA), which contributes to stress tolerance and detoxification processes (Seifi and Shelp, 2019; Chen et al., 2019). Additionally, polyamine metabolism has been shown to influence the expression of stress-responsive genes and interact with beneficial soil microbiota, thereby promoting plant health and resilience (Jiménez-Bremont et al., 2014). Recent advances also suggest that breeding and genetic approaches targeting polyamine biosynthesis and signaling pathways may provide new opportunities to improve crop tolerance to both biotic and abiotic stresses (Woo et al., 2014; Berg et al., 2016; Backer et al., 2018; Chen et al., 2019; Trivedi et al., 2020; Dunn and Becerra-Rivera, 2023). Key applications of PAs in crop protection and sustainable agriculture are summarized in **Table 4**. PAs offer diverse applications in modern agriculture through their integration into both biochemical and biological strategies. Exogenous applications such as seed priming and foliar treatments provide rapid enhancement of stress tolerance, while microbial and soil-based approaches contribute to long-term improvements in plant health and soil ecosystem stability. Furthermore, advances in genetic and microbiome engineering present promising opportunities to harness polyamine pathways for sustainable crop production and reduced reliance on chemical inputs. Despite the promising potential of polyamine-based approaches, several limitations remain before their widespread agricultural implementation. Most available evidence has been generated under controlled laboratory or greenhouse conditions, whereas comparatively few studies have evaluated their effectiveness under multi-location field environments (Backer et al., 2018; Trivedi et al., 2020). Furthermore, the optimal concentration, timing of application, and compatibility of PAs with different microbial inoculants are likely to vary among crop species, microbial communities, and environmental conditions (Chen et al., 2019; Kusano and Suzuki, 2015). A deeper understanding of interactions between polyamine metabolism, plant hormonal networks, and microbial community dynamics will therefore be essential for developing reliable polyamine-assisted biostimulants and integrated crop protection strategies. Future research should prioritize long-term field validation, formulation optimization, and integration with microbiome engineering approaches to facilitate the practical adoption of polyamine-based technologies in sustainable agriculture (Backer et al., 2018; Trivedi et al., 2020; Kusano and Suzuki, 2015).

**Table 4:** Major application strategies of polyamines in crop protection and sustainable agriculture, highlighting their mechanisms of action and potential benefits for improving plant resilience, productivity, and stress tolerance.

Application strategy	Method/approach	Target system	Mechanism of action	Agricultural outcome	Key references
Seed Treatment	Polyamine priming (Put, Spd, Spm)	Seeds/Seedlings	Activation of defense pathways, improved germination	Enhanced seedling establishment and disease resistance	Jiménez-Bremont et al., 2014; Seifi and Shelp, 2019; Wojtyla et al., 2024
Foliar Application	Exogenous polyamine sprays	Leaves/Shoots	Modulation of ROS, antioxidant activation	Improved tolerance to abiotic stress (drought, salinity)	Liu et al., 2015; Chen et al., 2019; Hasanuzzaman et al., 2020
Soil Amendment	Polyamine-enriched formulations	Rhizosphere	Interaction with soil microbiota, nutrient mobilization	Enhanced plant growth and soil health	Backer et al., 2018; Compant et al., 2019; Trivedi et al., 2020
Microbial Biocontrol Integration	Combination with PGPR/AMF	Plant–microbe system	Enhanced colonization, signaling crosstalk	Increased biocontrol efficiency and stress tolerance	Woo et al., 2014; Compant et al., 2019; Trivedi et al., 2020
Genetic Engineering	Overexpression of PA biosynthesis genes	Whole plant	Increased endogenous polyamine levels	Improved resilience to abiotic and biotic stresses	Liu et al., 2015; Chen et al., 2019; Takahashi, 2020
Microbiome Engineering	Manipulation of PA-producing microbes	Rhizosphere microbiome	Modulation of microbial community structure	Sustainable crop productivity and reduced chemical inputs	Berg et al., 2016; Compant et al., 2019; Trivedi et al., 2020

## 7 Methodological approaches and experimental considerations

PAs have emerged as key plant signaling molecules during interactions with beneficial and pathogenic microbes (Jiménez-Bremont et al., 2014; Seifi and Shelp, 2019). In monocots and dicots alike, PAs such as putrescine, spermidine, and spermine participate in almost every aspect of normal physiology and play critical roles in establishing and sustaining biotrophic, hemibiotrophic, and necrotrophic relationships with fungi or oomycetes (Chen et al., 2019; Takahashi, 2020). PAs modulate plant-microbe crosstalk in both beneficial and pathogenic contexts by influencing the expression of transcriptional regulators, metabolic enzymes, and hormones involved in prime signaling (Liu et al., 2015; Seifi and Shelp, 2019; Zhang et al., 2020). PAs also participate in the modulation of ROS and redox homeostasis and control the catabolism of PAs to produce signaling molecules such as reactive aldehydes and GABA of plant origin, as well as the release of the soil-dwelling, plant-protecting, polyamine-degrading fungus *Trichoderma* spp. in roots (Wang et al., 2011; Waszczak et al., 2018; Shao et al., 2022). The influence of PAs on non-microbe-related signaling is also sharply reduced during germination and early seedling development following exogenous application, emphasizing their involvement, during these early stages, in moderating the immunology of germination/damping-off diseases and cotyledon/micro-bean development (Jiménez-Bremont et al., 2014; Seifi and Shelp, 2019). Targeted or untargeted transcriptomic, proteomic, metabolomic, and fluxomic analysis, supplemented by lab-catalyzed GABAA-GABA-inspired and photo-reactive-based approaches, are recommended for the *in situ* study of crosstalk mechanisms (Wang et al., 2011; Trivedi et al., 2020; Zhang et al., 2020). The simultaneous application of data from all available omics analyses is essential for comprehensive understanding and visualization of crosstalk processes (Chen et al., 2019; Trivedi et al., 2020; Zhang et al., 2020).

### 7.1 Analytical techniques for polyamines and related metabolites

Quantitative analysis of PAs is essential for understanding their roles in plant–microbe interactions and stress responses. Because PAs occur at relatively low concentrations and possess similar chemical structures, sensitive analytical methods are required for their accurate identification and quantification. High-performance liquid chromatography (HPLC), often combined with pre-column derivatization using dansyl chloride or benzoyl chloride, remains one of the most widely used techniques for determining putrescine, spermidine, and spermine in plant tissues. Gas chromatography–mass spectrometry (GC–MS) and liquid chromatography–tandem mass spectrometry (LC–MS/MS) provide higher sensitivity and enable simultaneous profiling of polyamines together with related metabolites, including γ-aminobutyric acid (GABA), amino acids, and oxidative catabolites. Nuclear magnetic resonance (NMR)-based metabolomics has also been applied to characterize metabolic changes associated with PA metabolism under stress conditions. Beyond metabolite quantification, measurement of the activities of key enzymes involved in PA biosynthesis and catabolism—including arginine decarboxylase (ADC), ornithine decarboxylase (ODC), spermidine synthase (SPDS), spermine synthase (SPMS), and polyamine oxidase (PAO)—provides valuable information regarding metabolic regulation. Combining metabolite profiling with enzyme activity assays allows researchers to distinguish between altered biosynthesis, degradation, and transport of PAs during plant–microbe interactions. These analytical approaches provide the experimental basis for elucidating the molecular mechanisms underlying PA-mediated stress adaptation and crop resilience (Tavladoraki et al., 2006; Groppa and Benavides, 2008; Kusano et al., 2008; Chen et al., 2019; Takahashi, 2020).

### 7.2 Omics and systems biology approaches

Recent advances in omics technologies have substantially improved our understanding of PA-mediated plant–microbe interactions. Transcriptomic analyses (RNA-seq) enable the identification of genes involved in PA biosynthesis, catabolism, transport, and signaling during interactions with beneficial microorganisms and pathogens. Proteomic approaches reveal changes in enzymes associated with antioxidant defense, hormone signaling, and stress-responsive pathways, whereas metabolomics provides comprehensive profiles of PAs together with amino acids, phytohormones, and secondary metabolites involved in stress adaptation. Integration of transcriptomics, proteomics, metabolomics, and microbiome sequencing has facilitated systems-level analyses of PA-mediated regulatory networks. Multi-omics approaches enable reconstruction of signaling pathways connecting PA metabolism with ROS homeostasis, phytohormone crosstalk, and microbial community dynamics. More recently, single-cell transcriptomics, spatial metabolomics, and network biology have emerged as promising tools for resolving tissue-specific and cell-type-specific regulation of PA metabolism during plant–microbe interactions. Coupling these approaches with genome editing technologies and computational modeling is expected to accelerate the identification of key regulatory genes and facilitate the development of polyamine-assisted strategies for sustainable crop improvement (Cramer et al., 2011; Meena et al., 2017; Chen et al., 2019; Trivedi et al., 2020; Zhang et al., 2020; Shao et al., 2022).

### 7.3 Experimental design and data interpretation

The study of PAs in plants has gained significant momentum in the past two decades owing to the discovery of their involvement in signaling pathways under biotic and abiotic stresses (Moschou et al., 2008; Pál et al., 2015). PAs such as putrescine, spermidine, and spermine, which are cationic aliphatic amines, play important roles in cellular processes including cell division, differentiation, genome stability, gene expression, and membrane stability (Tavladoraki et al., 2006; Kusano et al., 2008). The modulation of polyamine metabolism by pathogens or beneficial microorganisms either in host or in the interacting partner has emerged as an important aspect of plant-microbe interactions (Moschou et al., 2008; Valdés-Santiago and Ruiz-Herrera, 2014). PAs have been associated with root exudates of various plants that interact beneficially with mycorrhizae or rhizobia or with non-pathogenic fungi like *Trichoderma* species against fungal pathogens (Vurukonda et al., 2016; Rouphael and Colla, 2020). Given the multifaceted roles of PAs during microbial encounters there is considerable interest in polyamine-mediated crosstalk and its applications in crop protection (Kusano et al., 2008; Pál et al., 2015). Investigation of free and conjugated PAs along with metabolic flux analysis can determine the extent of their contribution in different input scenarios (Kusano et al., 2008). The regulation of biosynthetic or catabolic pathways or their downstream effectors along with measurement of other commonly associated metabolites can help in unraveling specific involvement of PAs in plant-microbe interactions (Moschou et al., 2008). Polyamine pairing with transcriptomic, proteomic or metabolomic approaches enhances the understanding of crosstalk mechanisms besides allowing identification of polyamine-driven transcriptional regulatory circuits, signaling pathways, gene clusters or metabolite networks (Rouphael and Colla, 2020). Integration of polyamine profiling with plant resistance or tolerance traits can provide insights on their possible contribution in different input combinations (Pál et al., 2015; Vurukonda et al., 2016).

## 8 Challenges, gaps, and future directions

PAs play multiple roles in the interaction between plants and microbes (Jiménez-Bremont et al., 2014; Seifi and Shelp, 2019). They constitute a group of small aliphatic cationic molecules, being putrescine (Put), spermidine (Spd) and spermine (Spm) (Kusano et al., 2008; Chen et al., 2019). PAs are ubiquitous in biological systems and are synthesized from l-arginine and l-ornithine, which are intermediates of the arginine and urea cycles. Regardless of the pathways involved, the PAs produced are fundamentally the same throughout eukaryotes and are highly conserved in evolution (Kusano et al., 2008). PAs generally are present in larger amounts in plants than in other organisms. At the physiological level, PAs can be regarded as nutrients and serve as substrates for the synthesis of bioactive compounds. PAs control and regulate several important processes, including cell division, elongation and differentiation, senescence, root and shoot growth and organ development (Chen et al., 2019; Takahashi, 2020). They also modulate the synthesis of second messengers such as ROS, hydrogen peroxide, nitric oxide (NO) and cyclic adenosine mono phosphate (cAMP), thereby providing some flexibility and specificity to PA action, which is determined by the compartmentation, biochemical modulation and/or interaction with biochemicals at various cellular levels (Mittler, 2017; Seifi and Shelp, 2019). In addition to signaling, PAs may affect the metabolism of defense compounds such as phenolics, flavonoids, alkaloids, thionins, chitinase, β-1,3-glucanase and protease inhibitor genes (Shao et al., 2022). Microorganisms endeavor to colonize plant tissues to obtain nutrients and complementary elements essential for their development. Microbial pathogens exploit existing weaknesses in the plant defense system to establish an infection. Plant growth-promoting microorganisms are ubiquitous in nature. Their designation includes *Rhizobium*, Mycorrhizal fungi, Enterobacteria, Actinomycetes, *Azospirillum*, *Glomus* and *Azolla*, among others (Compant et al., 2019; Trivedi et al., 2020). PAs seem to participate throughout this spectrum. The exploitation of plant–microbe crosstalk co-opts microbiota to manipulate signaling networks. PAs occupy a strategic position in plant–microbe exchanges in systems under development to protect crops against threats to sustainability (Seifi and Shelp, 2019). They provide a key to improving resilience by exchanging information with beneficial microorganisms and enhance signal complexity and specificity (Chen et al., 2019; Trivedi et al., 2020). Fifty years ago, early studies showed that PAs may be involved in plant disease resistance; however, a strong agricultural payoff or broader conception of crosstalk remains elusive. Thus, the major focus here is centered on signaling rather than broader notions such as polyamine gradients or long-distance signaling.

## 9 Conclusion

The research reveals polyamine-mediated crosstalk across diverse plant–microbe interactions as a newly recognized mechanism for enhancing stress tolerance and crop protection. Characterizing the roles played by PAs in these systems sharpens insight into the underlying mechanisms and expands the repertoire of agronomically relevant traits that could be harnessed in microbial biocontrol systems and supported by microbial communities. PAs comprise low-molecular-weight aliphatic amines with widespread roles in all domains of life. In plants, PAs are (i) essential for growth, development and reproduction, (ii) a major component of the responses to both abiotic and biotic stresses, (iii) involved in the crosstalk of different stress-signaling pathways, and (iv) required, not only for normal growth, but also accumulate under stressful conditions. With the advent of comprehensive chemical stimulus identification, the broad-spectrum influences of PAs have been further illuminated. Recognition of their pivotal role in regulating plant processes has sparked further interest in the underlying mechanisms.
